# Patient Safety Domains in Primary Healthcare: A Systematic Review

**DOI:** 10.4314/ejhs.v34i1.9

**Published:** 2024-01

**Authors:** Hadi Kalantari, Pouran Raeissi, Aydin Aryankhesal, Seyyed Masoud Hashemi, Nahid Reisi

**Affiliations:** 1 Department of Health Services Management, School of Health Management and Information Sciences, Iran University of Medical Sciences, Tehran, Iran; 2 Department of Anesthesiology, School of Medicine, Shahid Beheshti University of Medical Sciences, Tehran, Iran; 3 Department of Pediatric, Hematology and Oncology, Isfahan University of Medical Sciences, Isfahan, Iran

**Keywords:** Patient safety, Primary healthcare, Medical errors, Adverse events

## Abstract

**Background:**

Healthcare systems should ensure the provision of quality services to patients without harming them. However, the provision of services is occasionally accompanied by harm or complications, most of which are preventable. Most studies have focused on secondary healthcare rather than primary healthcare (PHC). Thus, this study aimed to identify various dimensions and components of patient safety in PHC worldwide.

**Methods:**

This systematic review study was conducted in November 2022 based on PRISMA reporting guidelines. Studies were retrieved from PubMed, Scopus, Cochrane Library, Web of Science, and EMBASE and searched for English documents using the keywords “patient safety” and “PHC” from 2000 to 2022. Finally, two reviewers extracted the data independently and analyzed using thematic content analysis.

**Results:**

Overall, 23 out of the initially 4937 identified articles were selected for the final analysis based on the inclusion and exclusion criteria. Most of these studies used a qualitative-quantitative approach (61.9%, seven studies for both), and 64% had been conducted in European countries. Eventually, five dimensions and 22 components were identified for patient safety in PHC, including management measures, quality management, resources and technology, documents, and patient-related factors.

**Conclusion:**

The patient safety dimensions and components identified in this research can help develop a clear definition of patient safety and its assessment standards and criteria in PHC. Considering that most previous studies on patient safety in PHC were conducted in European and developed countries, it is suggested that researchers conduct more studies in developing countries to fill this research gap.

## Introduction

Countries with strong primary healthcare (PHC) mechanisms have more efficient health systems and better health outcomes than those focusing on hospital systems. Evidence suggests that strong PHC is accompanied by better public health, lower rates of unnecessary hospitalizations, and lower socioeconomic inequality. In addition, an advanced PHC system has several positive effects (e.g., better cost reduction opportunities and better health outcomes) on the health system (1, 2).

Although there are different types of PHC systems, not all have led to desirable outcomes. Over the past decade, technologically advanced countries have developed and used at least one method or system for primary care performance evaluation (3). Nonetheless, there is debate about the proper methods for collecting data on the quality and safety of PHC services (4, 5). Therefore, measuring PHC performance is necessary for evaluating health service outcomes, improving accountability, and guiding efforts at different levels of health systems (4).

Healthcare quality indicators measuring aspects of healthcare reveal the performance of healthcare service providers and/or healthcare systems. Experts use patient safety indicators to identify, monitor, and evaluate adverse events or dangerous conditions in healthcare that may cause undesirable health outcomes (6). As an indicator of healthcare quality, guaranteeing patient safety is important today. Accordingly, since the late 20th century, health communities have focused on the quality of care and discussed patient safety (7).

Extensive patient safety studies have been conducted in hospitals (8-10). Although most patients receive PHC services (11), few studies have examined the success of patient safety plans implemented in the PHC systems. Ensuring patient safety in the PHC system is undoubtedly considered a serious challenge. Estimating medical- or health-related error rates in primary care is difficult because, unlike hospital systems, PHC providers typically do not fully control healthcare management. Further, long delays in diagnosing and assessing patient safety incidents lead to challenges, and incomplete records can make it difficult to completely understand the suspected factors (12).

Nowadays, medical errors cause numerous cases of harm and/or deaths. In fact, medical errors have become a major problem for policy-makers, executive managers, and treatment and healthcare specialists because medical errors (e.g., medication errors, delayed referral of patients, and poor patient follow-up) occur frequently (9, 10, 13).

An essential requirement of a healthcare system is to provide patients with services that do not harm them. However, the provision of services is occasionally accompanied by harm or complications, most of which are preventable (5, 14). Furthermore, a 2011 report by the American Medical Association on patient safety demonstrated that although many people visit treatment and healthcare centers, insufficient research is conducted on patient safety in PHC than in secondary healthcare (15).

Adverse errors and events generally impose huge costs on the public and private sectors, families, and communities. Moreover, healthcare errors can have substantial impacts on a person's life. However, damage to the health system is often more extensive. For example, the National Health Service estimates the annual cost of medical errors to be £1-2.5 billion (16).

There is limited international evidence on how to effectively and sustainably improve patient safety in PHC. Therefore, a global movement on patient safety improvement in PHC has been launched to better understand the nature of medical errors, their outcomes, and how to cope with them (17).

The results of a study on PHC physicians in Riyadh, Saudi Arabia, revealed the high incidence of medication errors reported in PHC (18). However, the results of another study investigating the frequency of medical errors associated with electronic medical records in PHC centers in Kuwait indicated that 48% of healthcare providers rarely reported the occurrence of errors (19). Another study suggests that unsafe care endangers patients' lives, creates mistrust, and imposes enormous costs on the healthcare system (20).

The estimated incidence of significant harm in England primary care considered at least ‘probably’ avoidable is between 35.6 and 57.9 per 100,000 patient-years (the latter figure is based on sensitivity analysis). Extrapolating our findings to the English population of 55.6 million (mid-year 2017), it was found that there are likely to be between 19800 and 32200 cases of ‘probably avoidable’ significant harm to patients each year (21, 22). In Spain, about 3 million incidents occur annually in the PHC system. The most common PHC-related incidents in this country include problems with prescribing medications, exacerbation of clinical conditions, complications associated with medical procedures, and infections related to unsafe care (6).

In Brazil, the rate of healthcare-related incidents was approximately 1.11%, most of which were due to communication factors (23). In a systematic review, the most common PHC adverse events were related to medication and diagnostic problems. Communication problems among healthcare team members were identified as the main cause of these incidents (24).

Drastic changes must be made to improve safety at all healthcare system levels. The World Health Organization (WHO) formed a PHC expert group and published a four-section guide to provide those interested in PHC with the work of these distinguished specialists. This guide, which summarizes a series of technical studies, covers several areas, including patients (patient participation), healthcare personnel (education and training and human factors), care processes (administrative errors, diagnostic errors, medication errors, multi-morbidity, and transitions of care), and tools and technology (electronic tools).

Despite the increasing importance of patient safety in PHC, previous studies have rarely identified the dimensions and components of patient safety (clients of the health system) in PHC. Consequently, the current review aimed to identify various dimensions and components of patient safety in PHC worldwide.

## Materials and Methods

This systematic review was conducted based on the PRISMA-ScR reporting guidelines in November 2022.

**Data sources and search strategy**: Five electronic databases (PubMed, Scopus, Cochrane Library, Web of Science, and EMBASE) were systematically searched for relevant records published from 2000 to November 2022. To increase the comprehensiveness of the current systematic review, the reference list of related studies was also reviewed to identify more relevant articles. The most important search terms included “patient safety” and “primary healthcare”, along with their synonyms in medical subject headings (MeSH). An example of a search strategy in PubMed is as follows:

(“primary care” [tiab] OR “primary healthcare” [tiab] OR (Care [tiab] AND “Primary Health” [tiab]) OR (“Healthcare” [tiab] AND Primary [tiab]) OR (“Healthcare [tiab] AND Primary [tiab]) OR (Care [tiab] AND Primary [tiab]) OR “health center” [tiab]) AND (“patient safety” [tiab] OR “risk management” [tiab]).

This strategy has been defined and used for other databases based on the characteristics of each database. The search strategy of other databases is presented in Appendix 1. These searches were performed based on consultation with a medical library and information science specialist in November 2022.

**Inclusion and exclusion criteria**: The inclusion criteria were original and review articles related to patient safety in different countries, articles published in English, access to the full texts, and a time limitation of 2000-2022. On the other hand, articles that examined only patient safety in hospitals, case studies, letters, letters to the editor, editorial commentaries, comments, and conference articles were excluded from the study.

**Screening and data extraction**: The abstracts of all identified records were entered into EndNote x8. After removing duplicates, the titles and abstracts of all articles were screened, and those related to patient safety in PHC were identified accordingly. This process was conducted independently by two reviewers, and disputed cases were resolved by consulting with a third person. Finally, the full-text of the related studies was studied independently by two reviewers, and disagreements about including the full-text were resolved by consulting with a third person.

A data extraction form was developed and used for study characteristics and outcome data. The data such as the first author, the year of publication, the country, the purpose of the study, study design, the data collection method, participants, the sample size, and the domains of patient safety in PHC were extracted. The data extraction form was tested before the actual implementation by two reviewers independently.

**Critical appraisal of the included studies**: Joanna Briggs Institute checklists were utilized for assessing the quality of cross-sectional research (8 items) and qualitative studies (10 items). The SANRA (6 items with a total of 12 scores), and MMAT (6 items) scales were employed for quality assessment of literature reviews and mixed-methods studies, respectively. Studies with a score above 50% were included in the current review. The qualification of the evidence was conducted independently by two reviewers. In the case of disagreement, the third reviewer reviewed the article.

**Synthesis of results**: To analyze the data, qualitative and thematic content analysis methods were used based on Braun and Clark's model. The procedures included getting to know the data, creating primary codes, searching for semantic units in the text, reviewing semantic units, defining and naming semantic units, and reporting. Therefore, the domains of patient safety in PHC were determined as the main category, and the subcategories related to each aspect were identified from the reviewed studies. In addition, the overlapping cases were integrated, and the data were synthesized in MS Word 2016.

**Ethical considerations**: The study was approved by the Iran University of Medical Sciences, International Campus (IR.IUMS.REC. 1399.361).

## Results

**Figure 1:**
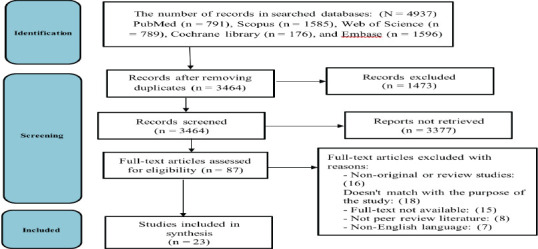
PRISMA flow diagram of studies retrieval

Figure 1 shows the process of selecting studies. Overall, 3,464 titles and abstracts and 87 full-text articles were screened after removing duplication. It was determined that 23 studies met the inclusion criteria. No additional studies were identified in the partial update search or reference list checking.

Table 1 presents the general characteristics of the selected articles. Most of these studies were conducted with a qualitative approach. Based on the findings, 16 of the eligible studies (64%) were conducted in European countries, while the remaining studies were undertaken in countries of South America (n = 4), Asia (n = 1), Australia (n = 1), and North America (n=l). The included studies used different designs such as qualitative (n = 7), mixed-method (n = 5) quantitative (n = 7), and review (n = 5) designs. The quality of the selected studies was assessed independently by two authors. All studies were of moderate-to-high quality.

**Table 1:** Characteristics of validation studies in the review

Author/year	Country	Study purpose	Research method	Participants	Data collection method	Quality assessment
Gaal et al., 2011 (2)	Austria, Denmark, France, Germany, the Netherlands, New Zealand, Slovenia, and the England	To identify the most important patient safety improvement strategies in primary care	Web-based survey	58 physicians and researchers	Questionnaire	4/8
Szecsenyi et al., 2011 (25)	Germany	To examine the effectiveness of the European practice assessment in improving management in primary care practices, with a focus on the domain of quality and safety	Before-after study	2014 practice manager and general practitioners	Questionnaire	7/10
Van Duimen et al., 2011 (26)	The Netherlands	To document patient safety in primary allied healthcare in the Netherlands and to identify factors associated with incidents	Retrospective study	1000 patient records	Prevention and recovery information system	8/11
de Bruin-Kooistra et al., 2012 (7)	The Netherlands	To identify a set of indicators for monitoring the quality of maternity care for low-risk women provided by primary care midwives and general practitioners	Delphi technique	28 midwives, 2 GPs, 3 obstetricians, and 3 maternity assistants	Questionnaire	6/8
Wammes et al., 2013 (27)	The Netherlands	To identify the most important organizational items in primary care that could be targeted by programs to improve patient safety	Web-based survey	65 physicians and researchers	Questionnaire	6/8
Bell et al, 2014 (28)	England	To produce a set of patient safety tools and indicators	Mixed method	Nine internationally-recognised experts	Literature review and expert panel	21/21
Alameddine et al., 2015 (4)	Lebanon	To assess the readiness of care providers in the PHC sector for the implementation of quality and patient safety indicators	Cross-sectional survey	943 clinical care providers	Questionnaire	6/8
Bowie et al., 2015 (14)	England and Ireland	To identify, develop, and build expert consensus on ‘good practice’ guidance statements to inform the implementation of safe systems for ordering laboratory tests and managing results in European primary care settings	Mixed method	GPs, practice nurses and practice managers, as well as patient safety researchers and clinical educators	Review, observation, focus groups, and workshops	8/10
Daker-White et al., 2015 (5)	England	To synthesize published qualitative research concerning patient safety in primary care in order to build a conceptual model	Meta-ethnography	Forty-eight studies	Review	10/11
De Vries et al., 2015 (29)	The Netherlands	How GP practices manage patient safety aspects related to point-of-care testing in everyday practice	Web-based survey	750 GP practices	Electronic questionnaire	6/8
Frigola-Capell et al, 2015 (6)	Spain	To present an international framework for patient safety indicators in primary care	Mixed method	Nineteen experts (family physicians, academics, management, and health policy advisors)	Review and modified Delphi survey	8/10
Hernan et al., 2015 (3)	Australia	To identify the factors that contribute to patient safety incidents in primary care	Qualitative study	34 patients	Focus group and interview	8/10
Ricci-Cabello et al., 2016 (30)	England	To explore patients' perceptions and experiences of patient safety in primary care	Qualitative study	27 primary care users	Focus group	9/10
Ricci-Cabello et al., 2017 (21)	England	To explore patients' experiences and perceptions of patient safety	Qualitative study	6736 primary care users	Open-ended questionnaire	8/10
Singh et al., 2016 (31)	USA	To discuss the global significance, burden, and contributory factors related to diagnostic errors in primary care	Narrative review	-	Review	6/6
Tudor Carl et al., 2016 (22)	England	To identify the main causes of and solutions to medication errors in primary care	Qualitative study	57 clinicians	Open-ended questionnaire	
Chaneliere et al., 2018(20)	France	To describe the underlying factors, specifically the human factors, that are associated with PSIs in PHC using CADYA	Mixed method	127 general practitioners	Focus groups and form	8/10
Ewald et al., 2018 (1)	England	To develop a set of quality indicators to assess and monitor pediatric primary care in Europe	Mixed method	Twenty-two of these pediatric experts	Systematic literature and consensus panel	16/21
Nora et al., 2019 (32)	Brazil	To identify the patient safety challenges described by health professionals in PHC	Scoping review	26 studies	Review	6/11
Fernholm et al., 2020 (33)	Sweden	To explore patients, who had experienced harm at the time of receiving PHC, and how primary providers and practice managers understood reasons for harm and possibilities to reduce the risk of harm	Inductive qualitative analysis	22 Patients	Structured questionnaire with free text answers	7/10
Gontijo et al., 2020 (17)	Brazil	To identify scientific production on safety-related aspects or characteristics in the performance of PHC professionals for professional safety constructs	Integrative literature review	16 articles	Review	7/11
Rocha et al., 2021 (23)	Brazil	To understand how patient safety actions are organized in the conception of PHC professionals	Qualitative approach	Two nurses and three dental surgeons	Online interviews	9/10
Silva et al, 2021 (34)	Brazil	To understand the perception of the PHC nursing team on patient safety	Qualitative approach	22 nursing professionals	Semi-structured interviews	9/10

Note: GP = General practitioneres; PSI = Patient safety incident; PHC = Primary healthcare; CADYA = Categorization of errors in primary care

Content analysis led to 320 codes from the obtained studies, which were categorized into five main themes and 22 sub-themes, the results of which are provided in Table 2.

**Table 2:** Dimensions and components of patient safety in PHC extracted from included studies

Themes	Sub-themes
**Management practices**	1-Leadership and management support (2-4, 6, 7, 17, 20, 21, 26, 28, 30, 32)
	2-Human resources (2, 3, 5, 7, 21, 26, 28, 29, 32, 34)
	3-Staff education and training (3-6, 14, 17, 21, 29, 32)
	4-Communications (2, 3, 5, 28, 32, 33)
	5-Continuitv of service (3, 4, 6, 14, 21, 32)
	6-Organizational culture and commitment (32)

**Quality management**	1-Presence of quality improvement systems (2, 6, 7, 25, 32)
	2-Final indicators for neonatal and maternal care (7)
	3-Safety culture (2-6, 14, 17, 21, 26, 28, 30, 32)
	4-Error management and reporting (2, 4, 6, 7, 21, 25, 28, 34)
	5-Infection control (17, 32)
	6-Detailed clinical procedures (32, 33)
	7-Clinical audit (32)

**Resources and technology**	1-Technology (2, 6, 20-22, 28)
	2-Resources and facilities (2-4, 6, 17, 20, 21, 25, 28, 29)
	3-Safe Daraclinical actions (6, 14, 21, 22, 29)
	4-Drug and vaccine management (2, 6, 22, 33)
	5-Medical Equipment (32)

**Documentation**	1-Proper documentation (2, 21, 33)
	2-Presence of guidelines (6, 21, 22, 25, 26, 33, 34)

**Factors related to patient**	1-Patient participation (2, 3, 5, 6, 17, 21, 22, 25, 26, 30, 32, 33)
	2-Patient education (3, 5, 20, 26, 28)

**Management practices**: The first theme that was identified from the data was management practices. This theme consisted of six sub-themes. Most studies reported that improving patient safety in PHC depends on management practices such as management and leadership support, human resources, staff education and training, communication, continuity of service, and organizational culture and commitment.

**Quality management**: Quality management factors such as quality improvement systems, final neonatal and maternal care indicators, safety culture, error management and reporting, infection control, detailed clinical procedures, and clinical audits were influential in promoting patient safety in PHC.

**Documentation**: Several studies indicated that documentation, including proper documentation and the presence of guidelines, effectively promotes patient safety in PHC.

**Resources and technology**: Resources and technology are essential for the implementation of PHC patient safety initiatives. This theme includes sub-themes such as technology, resources and facilities, safe preclinical actions, drug and vaccine management, and medical equipment.

**Factors related to patients**: The final theme identified from the studies was patient-related factors such as patient participation and patient education. Patients and their families can play an effective role in maintaining and improving patient safety through different roles. Additionally, patient education is considered a modality to mitigate patient safety risks.

## Discussion

The present study extracted and reported the results of 23 studies conducted on patient safety assessment in PHC. Based on the results, it seems that studies conducted on this issue do not have much history, and researchers and healthcare organizations at the national and international levels have recently paid attention to this issue, while quality and safety in PHC are not a new issue. Focusing on patient safety in PHC has increased in recent years. The reasons are the WHO's emphasis on the quality of PHC in the Astana statement in 2018 and the successful experience of patient safety-friendly hospitals, resulting in providing a PHC framework that is patient safety-friendly (35). Based on the results of the present study, the domains of patient safety assessment in PHC were categorized into five domains, including management measures, quality management, resources and technology, documentation, and patient-related factors. Further, the patient safety-friendly PHC framework introduced by the WHO has six domains: management and leadership, lifelong learning, patient and community involvement, a safe environment, and evidence-based safe care.

In their study, Dorosti et al. (2020), based on the opinions of experts, introduced six primary domains for assessing the safety of service recipients in PHC, including management and leadership, process management, audit of service recipient safety, human resources, involvement of service recipients, community involvement, and occupational safety (36). In one study conducted by Tabrizi et al. (2016) using the Delphi technique, a proposed model of clinical governance in the PHC system in Iran consisting of leadership (as a prerequisite dimension) had five primary dimensions, including quality management, community involvement, health information management, human resource development, and monitoring and evaluation (37). Based on many studies and the existing frameworks in this field and, unlike the existing safety frameworks in medical departments, occupational safety and the safety of health staff working in PHC have received less attention. PHC staff are considered the front line in dealing with all kinds of diseases. Thus, it is crucial to pay attention to the safety of the staff in this sector (2, 3, 34). Accordingly, it is recommended that health system policymakers pay special attention to the safety of PHC staff, service providers, and clients in this domain.

Among the domains identified in the patient safety framework in PHC are management and leadership, which were found in most of the patient safety frameworks extracted in this study. The commitment of top management is considered a basic prerequisite regarding patient safety and its evaluation by the existing frameworks in health organizations (32, 38). Moreover, attention should be paid to patient safety as a basic value in the organizational culture of PHC centers. In this regard, the management and leadership of the organization play a significant role (39). Thus, it is recommended that interventions should be designed and implemented to enhance the knowledge and familiarity of managers of each PHC unit about patient safety in these centers. It leads to increased familiarity of managers with patient safety and their commitment to improving patient safety in the organization (40). It is also recommended that improving patient safety should be considered as one of the performance evaluation criteria for the managers and staff of PHC centers. Quality management was the second domain identified in this study. The need to improve and manage quality and patient safety as the primary components of quality is addressed in this domain.

A patient safety-friendly organization emphasizes creating a care delivery system that seeks to prevent errors and learns from past errors based on a patient safety culture (4, 10). Thus, a management and information system for staff to learn from the errors that have occurred and the use of colleagues' experiences regarding patient safety can be highly useful in reducing common errors (8, 10). Documentation related to patient safety in care centers was another domain of patient safety in PHC extracted in the present study. Reputable national and international organizations in the field of health, including the Ministry of Health of different countries and the WHO, publish guidelines to improve the safety of patients. They should be provided to the health staff of these centers so they can use them if necessary (22, 41). In this regard, it is recommended that different countries design and implement an online system for easier access for staff by collecting documents and guidelines related to patient safety.

Another domain extracted from the articles included in this study was related to resources and technology. The advancement of technology in recent years has facilitated performing tasks and communicating. Therefore, due to the need to learn from the errors that occurred in the past, it is recommended that an error reporting system should be designed and implemented in PHC so staff can share their experiences with their colleagues in other health centers by reporting errors and referring to the reasons for the occurrence of such errors (25, 30). The last domain of patient safety in PHC identified from the reviewed articles was associated with patient-related factors. The involvement of patients or service recipients in PHC is crucial to ensure their safety. Patients who are aware of their safety have a more protective layer in preventing medical errors (42). Patients involved in their safety are more aware of the potential risks of the care they receive. These patients can better identify errors and play a significant role in the early detection and prevention of medical errors and adverse events. Hence, it is suggested that interventions should be designed and implemented to make patients familiar with potential errors that may endanger their health and safety in PHC centers.

To the best of our knowledge, this systematic review is the first to synthesize the evidence regarding dimensions of patient safety in PHC. The strengths of this review include the use of a comprehensive search strategy developed and peer-reviewed by librarians with expertise in systematic reviews. Moreover, all stages of the research (screening, quality appraisal, data extraction, and data analysis) were performed independently by two researchers to ensure its accuracy and consistency. However, there were several limitations to this review. First, there was marked heterogeneity among the reviewed studies. Second, a literature search was conducted in several electronic databases, while the grey literature was not searched. Thirdly, we included only articles published in English. Therefore, we may have lost valuable data on this topic.

Although researchers have paid more attention to the issue of patient safety in PHC in the last two decades, there is still no clear definition of patient safety and its assessment standards and criteria. It seems a clear definition of patient safety is a vital need at the present time and is a great help to those who want to design a model to assess and improve patient safety in PHC. The patient safety dimensions and components identified in this research can help achieve such objectives. Considering that most previous studies on patient safety in PHC were conducted in European and developed countries, researchers are suggested to conduct more studies in developing countries to fill this research gap.
